# An *Inositol 1*,*3*,*4*,*5*,*6-Pentakisphosphate 2-Kinase 1* Mutant with a 33-nt Deletion Showed Enhanced Tolerance to Salt and Drought Stress in Rice

**DOI:** 10.3390/plants10010023

**Published:** 2020-12-24

**Authors:** Meng Jiang, Yanhua Liu, Ruiqing Li, Shan Li, Yuanyuan Tan, Jianzhong Huang, Qingyao Shu

**Affiliations:** 1 National Key Laboratory of Rice Biology, Institute of Crop Sciences, Zhejiang University, Hangzhou 310058, China; mengjiang@zju.edu.cn (M.J.); 11616004@zju.edu.cn (Y.L.); lishan@zju.edu.cn (S.L.); tanyy@zju.edu.cn (Y.T.); jzhuang@zju.edu.cn (J.H.); 2 Hainan Institute of Zhejiang University, Yongyou Industry Park, Yazhou Bay Sci-Tech City, Sanya 572000, China; 3 College of Agronomy, Anhui Agricultural University, Hefei 230036, China; liruiqing@ahau.edu.cn; 4 Institute of Nuclear Agricultural Sciences, Zhejiang University, Hangzhou 310058, China

**Keywords:** genome editing, *OsIPK1*, phytic acid, rice, stress tolerance

## Abstract

*OsIPK1* encodes inositol 1,3,4,5,6-penta*kis*phosphate 2-kinase, which catalyzes the conversion of *myo*-inositol-1,3,4,5,6-penta*kis*phosphate to *myo*-inositol-1,2,3,4,5,6-hexa*kis*phosphate (IP_6_) in rice. By clustered regularly interspaced short palindromic repeats (CRISPR) and CRISPR-associated protein (Cas9)-mediated mutagenesis in the 3rd exon of the gene, three *OsIPK1* mutations, i.e., *osipk1_1* (a 33-nt deletion), *osipk1_2* (a 1-nt deletion), and *osipk1_3* (a 2-nt deletion) were identified in T_0_ plants of the rice line Xidao #1 (wild type, WT). A transfer DNA free line with the homozygous *osipk1_1* mutation was developed; however, no homozygous mutant lines could be developed for the other two mutations. The comparative assay showed that the *osipk1_1* mutant line had a significantly lower level of phytic acid (PA, IP_6_; −19.5%) in rice grain and agronomic traits comparable to the WT. However, the *osipk1_1* mutant was more tolerant to salt and drought stresses than the WT, with significantly lower levels of inositol triphosphate (IP_3_), reactive oxygen species (ROS) and induced IP_6_, and higher activities of antioxidant enzymes in seedlings subjected to these stresses. Further analyses showed that the transcription of stress response genes was significantly upregulated in the *osipk1_1* mutant under stress. Thus, the low phytic acid mutant *osipk1_1* should have potential applications in rice breeding and production.

## 1. Introduction

Phytic acid (PA), also identified as *Myo*-inositol-1,2,3,4,5,6-hexakisphosphate (IP_6_), is thought to be the main storage form of nutrient phosphorous (P) (~80%) constituting ~1.6% of the dry biomass in crop grains [1]. In seeds of most cereal, IP_6_ occurs as phytates in the protein bodies, which chelate with several divalent metal ions e.g., Ca^2+^, Zn^2+^, Mg^2+^, and Fe^2+^ [2]. During seed germination, endogenous phytase activity is induced and hydrolyzes the PA, releasing bound mineral cations, stored phosphorus (P), and inorganic phosphorus (Pi) that are utilized for seedling growth [3]. Due to the lack of phytase, monogastric animals cannot utilize PA and the micronutrients in phytates as a nutrient source [4]. Furthermore, the undigested phytic acid phosphorus (PA-P) in animal wastes has gradually become one of the main causes of P pollution [5]. Because of these PA-related environmental and nutritional adverse effects, mutagenesis and biotechnological approaches have been used to produce *low phytic acid* (*lpa*) mutant lines in cereals [6] such as maize [3,7], wheat [8], and rice [9,10,11,12,13].

Twelve genes predicted to encode enzymes in the PA metabolism pathway have been identified in rice seeds [14]. Mutations of these genes are recognized to create the phenotypes of LPA in cereals [5]. Inositol 1,3,4,5,6-penta*kis*phosphate 2-kinase 1 (IPK1) is reportedly to catalyze the final step in the biosynthesis of IP_6_, by which the inositol 1,3,4,5,6-penta*kis*phosphate (IP_5_) is further phosphorylated at position 2 to form IP_6_ [14,15,16,17]. The pathway of IP_6_ biosynthesis reported in *Saccharomyces cerevisiae* [17] and *Dictyostelium discoideum* share the last step: the phosphorylation of IP_5_ to IP_6_ by IPK1 designated as a 2-kinase enzyme. The *IPK1* mutant in *S. cerevisiae* presented a reduction in the capability to transfer mRNA from the nucleus and exhibited comprehensive incapability to synthesize PA. The *AtIpk1-1* gene was examined in *Arabidopsis* using T-DNA insertion mutant, and the IP_6_ level was decreased in the *AtIpk1-1* mutant by 83% [18]. Ali et al. [19] produced transgenic rice by down-regulating the *OsIPK1* expression using an RNAi-mediated technique. The reduction in IP_6_ content and concomitant increase in phosphate (Pi) level were found in transgenic rice plants with a seed-specific decrease in the gene expression of *OsIPK1*. The seed germination and some agronomic characteristics of transgenic rice were similar to the wild type (WT) [19]. These findings are consistent with earlier research indicating that the mutations of *IPK1* gene had no adverse effects on seed viability and agronomic performances in soybean [20,21].

The association of inositol phosphates in molecular and cellular responses of plants to abiotic stresses has been investigated. For instance, IP_3_ was found to be transiently induced in plants under abscisic acid and salt stress [22,23]. The physiological and molecular proof of the role of IPK1 in the response to abiotic stresses is vague, although inositol phosphates are recognized as signaling molecules in the response to stresses. The clustered regularly interspaced short palindromic repeats (CRISPR) and CRISPR-associated protein 9 (Cas9), which is a precise and efficient approach for genome editing [24,25,26], has been used in crop breeding, particularly for generating *low phytic acid* (*lpa*) rice. In this study, we generated *osipk1* mutants by CRISPR/Cas9-mediated mutagenesis and analyzed seed accumulation of phosphorus (total phosphorus, TP; phytic acid phosphorus, PA-P; inorganic phosphorus, Pi), agronomic traits, seed germination, and stress tolerance with the aim to evaluate and explore the probability of producing the LPA rice with no adverse effect on agronomic traits and seed viability, and with better tolerance to salt and drought stresses.

## 2. Results

### 2.1. Mutations of *OsIPK1* and Development of Homozygous Transgene-Free Mutant Lines

**Figure 1:**
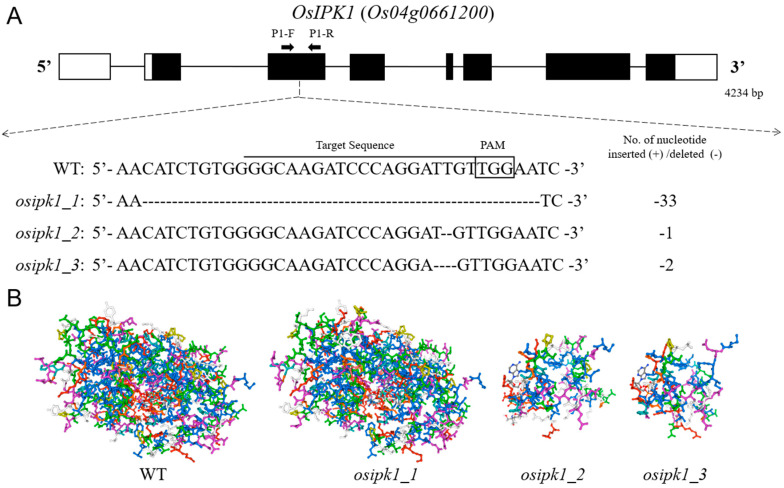
Schematic diagram of *OsIPK1* and the sgRNA target site for its clustered regularly interspaced short palindromic repeats (CRISPR) and CRISPR-associated protein (CRISPR/Cas9)-mediated mutagenesis (**A**), and prediction of IPK1 proteins in Xidao #1 (WT) and its *osipk1* mutants (**B**). Exons, introns and untranslated regions (UTRs) are indicated by solid boxes, lines and blank boxes, respectively. P1-F and P1-R are primers for genotyping *osipk1* mutations, with their position indicated by arrowheads. The protospacer adjacent motif (PAM) sequences (NGG) are boxed and the 20-nt target sequences are underlined. ‘–’ indicates deletion of nucleotide. The 3-dimensional structures of WT and mutant *osipk1* were analyzed on SWISS-MODEL (https://www.swissmodel.expasy.org/).

A homozygous *osipk1_1* mutant line with a 33-nt deletion in the third exon of the gene was generated by CRISPR/Cas9-mediated mutagenesis. Twenty-one hygromycin phosphotransferase (HPT)-positive T_0_ rice seedlings were selected from transformation with the vector of CRISPR/Cas9, pH_ipk1, and eight rice seedlings were finally verified mutated at the position of target. This signifies an editing efficiency of 38.1%, which manifests that the pH_ipk1 is efficient. Eventually, one homozygous transgene-free *osipk1* mutant (*osipk1_1*) and two heterozygous transgene-free *osipk1* mutants (*osipk1_2* and *osipk1_3*) (Figure 1A) were selected in T_0_ plants. Rice seeds of the mutated T_0_ seedlings were harvested and planted as lines of T_1_ plants. Interestingly, we found that no presence of homozygous transgene-free in T_1_ plants of *osipk1_2* and *osipk1_3*, whereas we can test some homozygous *osipk1_2* and *osipk1_3* mutations in the seeds which were not germinated. The same findings also appeared in the T_2_ and advanced-generation plants, so the heterozygous *osipk1_2* and *osipk1_3* mutants could not be used for further analysis. This may be due to a serious decline in phytic acid content in these IPK1 knock-out plants, which prevented seeds from germinating. Approximately ten surviving seedlings of the T_1_ line of *osipk1_1* were verified for the presence of target mutations and T-DNA at the seedling stage after bentazon treatment. All T_1_ seedlings of the selected line were further confirmed for both the presence of mutation and T-DNA. Rice seeds from the confirmed homozygous, transgene-free mutant seedlings selected as the mutant line *osipk1_1*, were harvested and used for further evaluation.

The mutation of *osipk1_1* (a 33-nt deletion) would cause an 11-amino acids fragment missing from amino acid positions 65 to 75 (Figure S1). Investigation of IPK1 proteins in nine organisms implied that the loss of 11-amino acids in the *osipk1_1* mutant was positioned in a highly conserved segment (Figure S2), signifying that the *osipk1_1* mutation could have potential functional consequences. In contrast, other mutations, *osipk1_3* (a 2-nt deletion) and *osipk1_2* (a 1-nt deletion) would produce a premature stop codon almost right after the site of mutation (Figure S1). Consequently, the mutant alleles of *osipk1_3* and *osipk1_2* were predicted to generate proteins with only 75 and 89 amino acids (Figure 1B and Figure S1). This may be the major reason why the homozygous transgene-free *osipk1_2* and *osipk1_3* mutants could not germinate.

### 2.2. Plant Growth and Phosphorus Content of *Osipk1_1* Mutant

**Figure 2:**
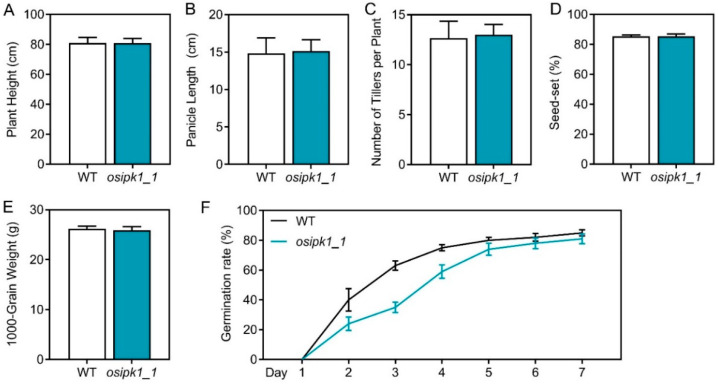
Agronomic traits (**A**–**E**) and seed germination rate (**F**) of Xidao #1 (wild type, WT) and its *OsIPK1* mutant line *osipk1_1*. Twenty single plants were investigated for WT and *osipk1*_1. Error bars represent standard error. (**F**) The germination rate was recorded from 1 to 7 days after soaking, and three replicates in each group.

Plant growth of *osipk1_1* was comparable with WT. No significant differences in the plant height, panicle length, tiller numbers per plant, seed-set, and 1000-grain weight were observed between Xidao 1 and *osipk1_1* (Figure 2A–E). The seed germination rate of *osipk1_1* was lower in the first four days, but caught up with that of the WT after five days, and almost the same on the 7th day (Figure 2F).

**Figure 3:**
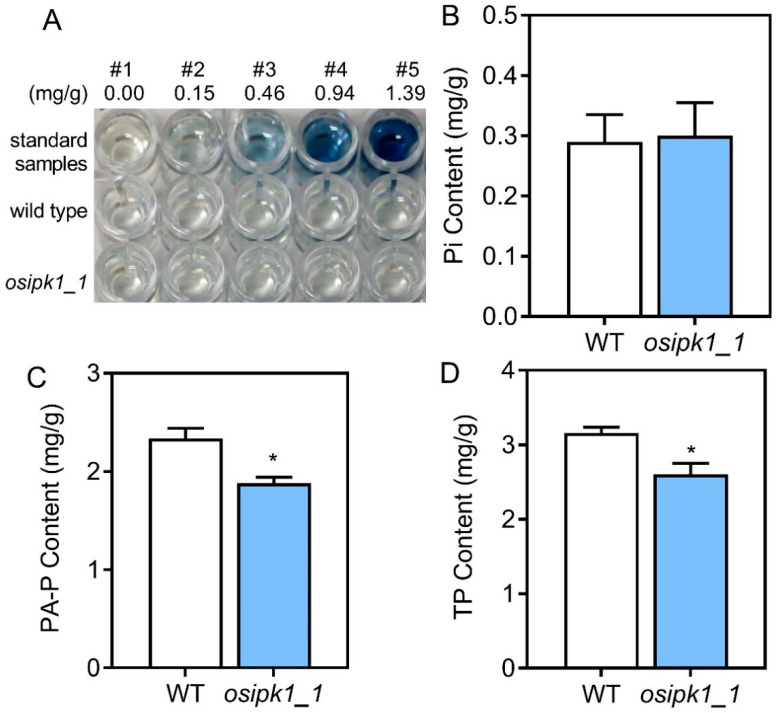
Inorganic P (Pi), phytic acid P (PA-P), and total phosphorus (TP) contents in Xidao #1 (WT) and its *OsIPK1* mutant line *osipk1_1*. (**A**) Qualitative assay of inorganic phosphorus (Pi) in mutant seeds. The concentration of the Pi standard samples were shown above. Five replicates were performed for WT and *osipk1*_1. (**B**–**D**) Three replicates were performed for WT and *osipk1*_1. Error bars represent standard error. Data with an asterisk(s) are significantly different from WT (* *p* < 0.05).

The colorimetric assay showed that *osipk1_1* had a similar Pi level to the WT control (Figure 3A). To measure the mutational effects of *osipk1_1,* the contents of Pi, PA-P, and TP were evaluated in seeds between the mutant line and WT. The *osipk1_1* mutant line had greatly lower PA-P and TP content compared with the control, while the content of Pi was not greatly different from that of WT (Figure 3B–D). The seeds of WT had a Pi level of 0.29 mg/g, which was not significantly different from the *osipk1_1* mutant line (Figure 3B). *osipk1_1* had a PA-P level of 1.88 mg/g, which was 19.5% lower than the control (2.34 mg/g) (Figure 3C). *osipk1_1* had TP content of 2.60 mg/g, which were 17.6% lower than the control (3.16 mg/g) (Figure 3D).

### 2.3. Mutant of *Osipk1_1* Has a Better Tolerance against Drought or Salt Stress than Wild Type (WT)

**Figure 4:**
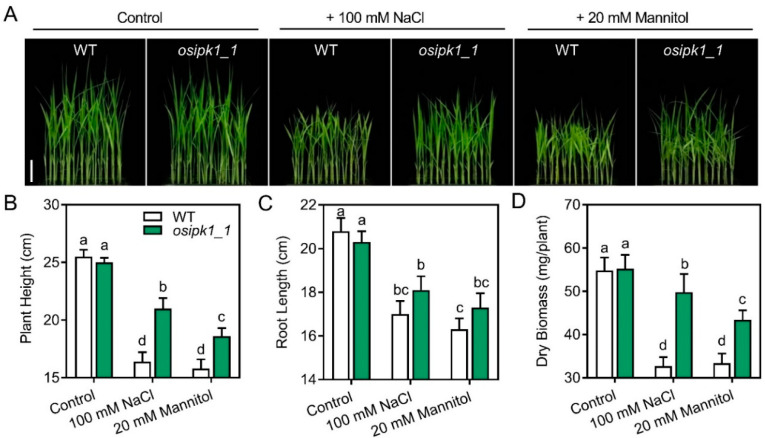
The phenotype of Xidao #1 (WT) and its *OsIPK1* mutant line *osipk1_1* under salt stress (100 mM NaCl) and drought stress (20 mM mannitol). (**A**) The picture was taken in the 7th day after treatment. Bar = 5 cm. (**B**–**D**) Six replicates were performed for WT and *osipk1_1*. Error bars represent standard error. The different letters (a, b, c, and d) show the significant difference at a probability of *p* ˂ 0.05.

To verify whether the mutant of *osipk1_1* also has any effect on stress tolerance, we tested the *osipk1_1* and WT seedlings to salt and drought stress treatment. The growth of *osipk1_1* seedling was similar to that of the WT when grown under normal conditions, while better than the control under either salt (100 mM NaCl) or drought (20 mM mannitol) stress conditions (Figure 4A).

After 7-day treatment by 20 mM mannitol or 100 mM NaCl, the plant height, root length, and dry biomass of WT seedlings were greatly decreased (Figure 4B–D). In contrast, the plant height, root length, and dry biomass of *osipk1_1* were significantly higher than that of WT plants after stress treatments (Figure 4B–D), signifying that the mutant of *osipk1_1* is much more tolerant against drought or salt stress than WT seedlings.

### 2.4. The *Osipk1_1* Mutant Accumulated Less Reactive Oxygen Species (ROS) than WT under Salt and Drought Stresses

**Figure 5:**
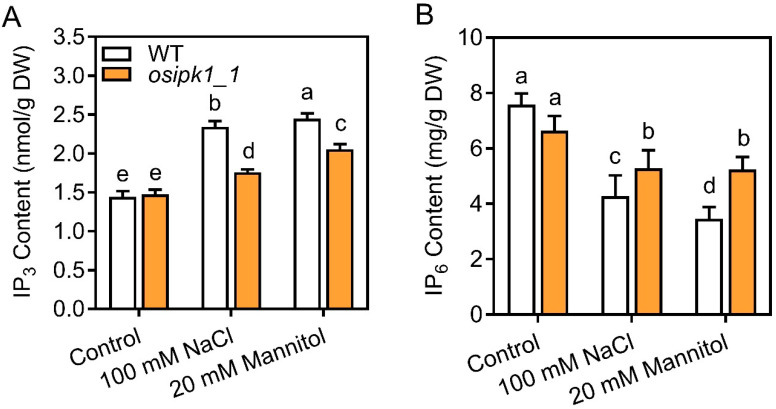
Accumulation of inositol triphosphate (IP_3_, **A**) and phytic acid P (IP_6_, **B**) in Xidao #1 (WT) and its *OsIPK1* mutant line *osipk1_1*. All analyses were performed with three replicates. Error bars represent standard error. The different letters (a, b, c, d, and e) show the significant difference at a probability of *p* ˂ 0.05.

**Figure 6:**
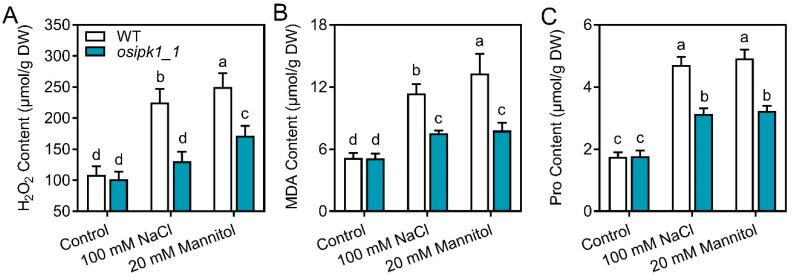
Levels of hydrogen peroxide (H_2_O_2_, **A**), malondialdehyde (MDA, **B**), and proline (Pro, **C**) in Xidao #1 (WT) and its *OsIPK1* mutant line *osipk1_1*. All analyses were performed with three replicates. Error bars represent standard error. The different letters (a, b, c, and d) show the significant difference at a probability of *p* ˂ 0.05.

**Figure 7:**
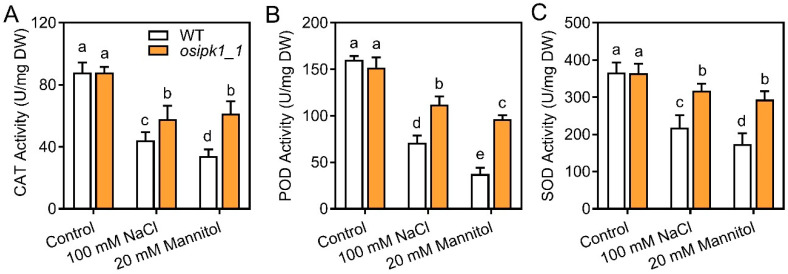
Activity of catalase (CAT) (**A**), peroxidase (POD) (**B**) and superoxide dismutase (SOD) (**C**) in Xidao #1 (WT) and its *OsIPK1* mutant line *osipk1_1*. All analyses were performed with three replicates. Error bars represent standard error. The different letters (a, b, c, d, and e) show the significant difference at a probability of *p* ˂ 0.05.

To explore how mutation of *osipk1_1* alleviated salt and drought stress, we measured the contents of IP_3_, IP_6_, stress-related free amino acid proline (Pro), ROS (H_2_O_2_), malondialdehyde (MDA), and anti-oxidant enzyme activities (peroxidase (POD), catalase (CAT), and superoxide dismutase (SOD)) in seedlings subjected to stresses (Figure 5, Figure 6 and Figure 7). Without treatment, all these parameters were not significantly different between WT and *osipk1_1*.

Firstly, we found that the level of IP_3_ was significantly increased after salt or drought stress treatment in WT, i.e., by 1.62-fold with salt stress, and by 1.69-fold with drought stress, respectively, relative to control after treatment for seven days (Figure 5A). The content of IP_3_ was also significantly increased in *osipk1_1*, but was still lower than that in WT after one-week treatment of salt or drought stress (Figure 5A). We also observed that seven days of stress treatment greatly reduced the accumulation of IP_6_ in WT (Figure 5B). In contrast, a significantly increased level of IP_6_ was observed in *osipk1_1* compared to WT (Figure 5B).

Secondly, we found that the accumulation of Pro, H_2_O_2_, and MDA were significantly increased after salt or drought stress treatment in WT (Figure 6). Following a one-week salt or drought stress treatment, the contents of Pro, H_2_O_2_, and MDA were also elevated in the mutant of *osipk1_1*, although with less magnitude as those of WT (Figure 6).

Thirdly, we found that seven days of stress treatment greatly decreased the anti-oxidant enzymes (POD, CAT, and SOD) activities, relative to control in WT (Figure 7). Following seven days of stress treatment, the anti-oxidant enzymes activities of CAT, POD, and SOD were also decreased in the mutant of *osipk1_1*, while with less magnitude compared those of WT (Figure 7).

These findings exhibited that *osipk1_1* decreased the ROS level and mitigated oxidative stresses with induced antioxidant enzyme activities and decreased level of IP_3_, compared to WT, under drought and salt stresses.

### 2.5. Transcription of Stress Response Genes Was Significantly Upregulated in the Osipk1_1 Mutant under Stress

To explore the molecular mechanism of how *osipk1_1* enhanced stress tolerance, the analysis of gene expression was conducted for phytic acid biosynthetic genes and stress-related genes in rice plants. 

Mutant of *osipk1_1* had a significantly higher abundance of phytic acid biosynthesis genes (except for *OsIPK1*) in the absence of stress treatment (Figure S3). Gene expression of *OsIPK2*, *OsITPK1*, *OsITPK2*, *OsITPK3*, and *OsITPK6* were significantly induced, while *OsIPK1*, *OsITPK4*, and *OsITPK5* expression were significantly decreased in WT after treatment (Figure S3). In *osipk1_1*, the abundance of *OsIPK2*, *OsITPK1*, *OsITPK2*, *OsITPK3*, and *OsITPK6* were significantly lower and *OsIPK1*, *OsITPK4*, and *OsITPK5* were significantly higher compared to WT seedlings after treatment (Figure S3).

**Figure 8:**
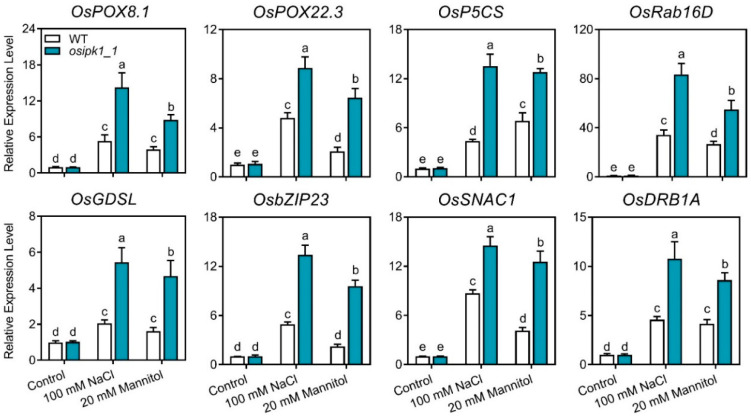
Relative expression level of stress related genes in Xidao #1 (WT) and its *OsIPK1* mutant line *osipk1_1*. All analyses were performed with three replicates. Error bars represent standard error. The different letters show the significant difference at a probability of *p* ˂ 0.05. The stress related genes encode various proteins including two peroxidase genes *OsPOX8.1* and *OsPOX22.3*, proline synthesis protein *OsP5CS*, rab16 family protein *OsRab16D*, osmolytes metabolism enzyme *OsGDSL*, bZIP transcription factors *OsbZIP23*, NAC (NAM/ATAF/CUC) transcription factor *OsSNAC1* and dehydration responsive element-binding protein *OsDREB1A*.

The expression of stress responsive genes was not significantly different between WT and *osipk1_1* without treatment (Figure 8). Exposure to salt and drought stress greatly induced the transcription of all eight tested genes (*OsPOX8.1*, *OsPOX8.1*, *OsP5CS*, *OsRab16D*, *OsGDSL*, *OsbZIP23*, *OsSNAC1*, and *OsDRB1A*) in WT (Figure 8). Furthermore, *osipk1_1* had a significantly increased abundance of these stress-response genes with salt and drought treatment compared to WT seedlings (Figure 8).

These results demonstrated that the expression of phytic acid biosynthetic genes was deregulated and stress-response genes were significantly up-regulated in *osipk1_1* mutant under salt and drought stress conditions.

## 3. Discussion

The CRISPR/Cas9 system has been broadly utilized in plant functional genomics studies. Our study intended to offer such an instance on the manipulation of PA content in rice grains by targeted mutagenesis of a PA synthesis gene. To develop rice genetic resources with agronomically competitive LPA, we targeted mutagenesis of *osipk1* using CRISPR/Cas9-mediated genome editing technology in this research. The homozygous and transgene-free mutant, named as the mutant line of *osipk1_1*, was evaluated for its seed P level together with the WT. The mutant line had significantly lower PA-P and TP contents compared with the control, while the Pi had no significant difference compared to the WT (Figure 3). *IPK1* is believed to catalyze the last step in inositol metabolism by using the inositol 1,3,4,5,6-pentaphosphate as the substrate [14,15,16,17]. This process only adds one phosphate group, which may result in a significantly decreased level of phytic acid, while the Pi content of *osipk1_1* does not significantly change. By contrast, the Pi level of transgenic rice was increased and the TP was similar to the WT [19]. In our present study, the agronomic traits and seed germination rate of *osipk1_1* were not significantly different from those of the WT, which was similar to the results of Ali et al. [19]. It was reported that seed-specific downregulation of *OsIPK1* did not negatively affect agronomic traits, but increased tolerance to artificial aging and seed viability [19]. The findings implied that the editing of the *OsIPK1* gene in rice seeds might be a valid way to generate LPA rice.

Terrestrial plants are frequently faced with numerous abiotic stresses throughout their whole life. Physiological, biochemical, and molecular responses to such stresses are mediated by a group of signal transduction pathways. Former studies have found that IP_3_ can be a vital second messenger, which always displays a transient induction upon exposure to exogenous stimuli, e.g., pathogens, reactive oxygen, light, osmotic, and salt stresses [23,27,28]. The relationship between abiotic stresses and metabolism of IP_3_ has been observed in mammalian cells and yeast (*Saccharomyces cerevisiae*) [22,23,29], but very little research has been described regarding the association between abiotic stresses and inositol phosphates in terrestrial plants. In tomato plants, genetically decreasing IP_3_ by increasing InsP_3_ hydrolysis caused enhanced drought stress tolerance [30]. The content of IP_3_ in *osipk1_1* was increased by salt and drought stresses in WT and showed a reduced level in *osipk1_1* (Figure 5A). Delineation of the stress-insensitive phenotype of *osipk1_1* would help understand the stress signaling pathway leading to less accumulation of IP_3_ and a non-standard level of a secondary messenger.

Free proline has been considered to contribute to the protection of macromolecules during dehydration [31] and the osmotic adjustment [32] as well as to be an important scavenger of hydroxyl radical [33]. The free proline content was induced following stress treatment but significantly lower in *osipk1_1* (Figure 6C). A similar change was observed for MDA (Figure 6B), which is an important intermediate in ROS scavenging under abiotic stress, and is toxic to the plant cells if over accumulated [34,35]. The activities of antioxidant enzymes can be inducible by oxidative stresses produced by salt and drought stresses. In this study, however, POD, CAT, and SOD activities were lower after treatment of 100 mM NaCl and 20 mM mannitol (Figure 7). Phytic acid is a natural antioxidant in plants, constituting 1–5% of most legumes, nuts, oilseeds, cereals, spores, and pollen [36,37]. Although IP_6_ was significantly reduced by salt or drought stress in both WT and mutant, a significantly higher level occurred in *osipk1_1* (Figure 5B). It is inferred that more accumulation of IP_6_ will alleviate the stresses of salinity and drought. The decreased activities of antioxidant enzymes and phytic acid may have resulted from the oxidative burst in plants under salt and drought stresses as revealed by the ROS level, e.g., H_2_O_2_ (Figure 6A) in stressed plants. These oxidative stresses appeared to be greatly alleviated in *osipk1_1* (Figure 6), signifying that improvement of the capacity of anti-oxidant defense is the main mechanism for mitigation of abiotic stress toxicity in rice seedlings of *osipk1_1*. Our findings propose that *osipk1_1* had improved capacity for ROS scavenging and osmotic adjustment.

We further examine whether the expression of *OsIPK2* and the other six ITPK members in rice have any changes in *osipk1_1* under salt or drought stress. The OsITPK genes’ exon-intron organization structures suggest that three ITPKs in rice (OsITPK1, OsITPK2, and OsITPK3) belong to subgroup I, with each including 9 introns and 10 exons with similar phases [38]. OsITPK4 and OsITPK5 included no intron and belong to subgroup II. OsITPK6, belonging to subgroup III, have 11 introns and 12 exons. The analysis of the expression level proposes that the strictly related OsITPKs have small differences in the patterns of gene expression in their responses to various stresses.

Previous studies showed that *OsPOX22.3* and *OsPOX8.1*, both encoding plastidic peroxidases, are involved in the tolerance to oxidative stress [39]. *OsP5CS* (*LOC_Os05g38150*) encodes an important enzyme for biosynthesis of proline and *OsGDSL* (*LOC_Os02g57110*), a GDSL-like lipase gene, was increased by osmotic stress [40]. *OsSKIPa* (*LOC_Os02g52250*), *OsbZIP23* (*LOC_Os02g52780*), *SNAC1* (*LOC_Os11g03300*), and *OsDREB1A* (*LOC_Os09g35030*), all being transcription factor genes, were also reported to be responsible for the tolerance to oxidative stress [41,42,43,44]. The gene expression levels of the above genes were greatly higher in *osipk1_1* than that in the WT under abiotic stress. These findings imply that the transcription factors above are associated with the downstream manipulation of the responses to abiotic stress in *osipk1_1*.

Since there is only one tested mutant of *osipk1* in our research, its excellent performance may be caused by the 33-nt deletion of *OsIPK1*, but it may also be due to accidental occurrence during the material cultivation process, e.g., tissue culture-derived variation, transposon (*Tos17*) insertion mutagenesis, pleiotropy. Firstly, antibiotics, like hygromycin in our research, might also enhance variation frequency [45,46], and thus several round screenings of calli on hygromycin-containing media might significantly induce mutations in rice. Second, to generate transgenic plants by using *Agrobacterium* transformation spent more time than regular tissue culture, while it has been well documented that the longer the tissue culture time, the higher the somaclonal variation frequency, because the somaclonal variation occurred throughout the culture process [47]. Therefore, further studies are needed to ascertain the real causative factor(s) leading to the enhanced tolerance of *osipk1_1* to salt and drought stresses. For example, progenies derived from a cross between *osipk1_1* and its WT parent could be used for such purposes.

In conclusion, by targeted mutagenesis of the *inositol 1,3,4,5,6-pentakisphosphate 2-kinase* gene using the CRISPR/Cas9 system, we generated *osipk1* mutant lines. Physiological, biochemical, and molecular data showed that the *osipk1_1* mutant had a better tolerance to salt and drought stresses. While the genetic cause remains undetermined, the enhanced tolerance of *osipk1_1* might be exploited in rice breeding.

## 4. Materials and Methods

### 4.1. Generation of Mutants Using Clustered Regularly Interspaced Short Palindromic Repeats (CRISPR) and CRISPR-Associated Protein (Cas9)

To produce *osipk1* mutants, the target site was chosen for *OsIPK1* (*LOC_Os04g56580/Os04g0661200*) in the third exon (Figure 1A). The UniProt (http://www.uniprot.org/) was used for target selection to minimize the off-target effect [48] and the sgRNAs for the precise site were designed by the CRISPR-P 1.0 website (http://crispr.hzau.edu.cn/CRISPR/). DNA base pairing sequence (C1-F and C1-R, Table S1) was synthesized by Tsingke (Hangzhou, China) for the construction of the vector of CRISPR/Cas9 (pH_ipk1) carrying an element of CYP81A6-hpRNAi using the plasmid of pHUN4c12s [49], which was improved from pHUN4c12 [50]. The plasmid pH_ipk1 was created and transformed into *Agrobacterium tumefaciens* and introduced into rice calli induced from the mature seed of ‘Xidao 1′ (a *japonica* rice cultivar) according to Li et al. [51].

### 4.2. Growth and Identification of Mutants

The transgenic plantlets were regenerated from *hygromycin*-resistant calli and acclimatized in a growth chamber (a photoperiod of 12 h, 30 °C) for seven days before being moved into outdoor facilities. Total genomic DNA was extracted from leaves of transgenic T_0_ seedlings according to a modified method of cetyltrimethylammonium bromide (CTAB) [52]. The existence of the gene of *hygromycin phosphotransferase* (*HPT*) was tested by polymerase chain reaction (PCR) amplification using the primer pairs HygR-F and HygR-R (Table S1) [53]. The site-specific mutation was identified by amplification of PCR using primers (P1-F and P1-R, Table S1) encompassing the target sites in the gene of *OsIPK1* (Figure 1A). The analysis of high resolution melt (HRM) was carried out according to Li et al. [54] to verify for the mutations. The fragments of a selected target with diverse colored lines on analysis of HRM were sequenced by Tsingke (Hangzhou, China) and the DSDecode (http://skl.scau.edu.cn/dsdecode/) program was used to decode the mutation sequences [55].

### 4.3. Development of Transgene-Free Mutant Lines

T_1_ plants were foliar sprayed with 1.0 g·L^−1^ bentazon (0.1 L·m^−2^) at approximately four-leaf stage according to Lu et al. [49]. Because CYP81A6, which metabolizes and renders tolerance to bentazon was silenced in the transgenic plants, the transgene-free plants will survive the betazone spraying and the transgenic ones will die. No less than 10 survived T_1_ plants from every single T_0_ plants were further verified for the presence of site-specific mutations and absence of T-DNA. The T-DNA free *osipk1* mutants were advanced to T_2_ lines and used for further tests.

### 4.4. Assay of Agronomic Traits

Xidao #1 (WT) and its transgene-free *osipk1* mutant were grown side by side at the facility of Zhijiang Seed Tec. Ltd., Hangzhou, Zhejiang, China. Sixty seedlings were planted for each line, and 20 randomly chosen inner plants of each plot were assessed for agronomic traits in the field, recorded before and after harvest. Different agronomic parameters, such as 1000-grain weight, seed-set, panicle length, number of tillers per plant, and plant height were evaluated.

### 4.5. Assay of Seed Germination

For a controlled germination test, the rice seeds were immersed in distilled water at 28 °C for two days and germinated on filter paper immersed with ultrapure water for seven days in darkness at 28 °C. The percentage of germination was noted at regular intervals and assayed. The tests were repeated thrice with 100 seeds for each repeat.

### 4.6. Assay of Seed Phosphorus

The contents of inorganic P (Pi) in seeds were examined both quantitatively and qualitatively by following the protocol of Chen et al. [56] with slight modifications. The qualitative assay was utilized for observation of the high inorganic P (HIP). The seeds of rice were placed into 96-well plates, mixed with the solution of 0.4 M HCl at 4 °C for 12 h. Aliquots of supernatant (10 μL) were used for the determination of Pi content by following the method of Larson et al. [9]. The colorless samples characterize contents of parent varieties, while the change of a blue color infers induced content of Pi (HIP) (Figure 3A).

The levels of Pi in rice seeds were quantitatively measured by following the method of Wilcox et al. [57] with minor modifications in triplicate. The grains of brown rice were pestled into flour and approximately 0.5 g flour was mixed with trichloroacetic acid including 25 mM MgCl_2_, 12.5 % (*w*/*v*) by gentle shaking at 4 °C for about 12 h. The supernatants were utilized for the assay of Pi by following the method of Raboy et al. [58] after centrifugation for 15 min at 10,000× *g*.

The accumulation of PA-P was measured for flour of brown rice by following the protocol of McKie et al. [59] using the commercial assay kit (Megazyme, Ireland) in triplicate. Approximately 1 g grains of brown rice was mixed with 20 mL of 0.66 M hydrochloric acid at room temperature for 2 h and then centrifuged for 20 min at 12,000× *g*. Immediately, the supernatant (0.5 mL) was neutralized by the addition of the same volume solution of 0.75 M sodium hydroxide. The mixed sample extract was used in the procedure of enzymatic dephosphorylation reaction and the absorbance was measured at 655 nm.

The contents of total phosphorus in rice seeds were measured by following the method as previously described in triplicate [60]. Approximately 0.2 g samples of brown rice were digested with 6 mL HNO_3_ for 60 min at 160 °C in a system of microwave digestion (Mars6, USA). The solution was concentrated for about 120 min at 140 °C until 1 mL digested solution was left, and then diluted into 50 mL distilled water [61]. Inductively coupled plasma mass spectrometry (ICP-MS) (PerkinElmer, USA) was used to analyze the total phosphorus in rice seeds.

### 4.7. Stresses Treatment

For the treatment of stresses, 14-day-old rice plants were grown in ½ Murashige and Skoog (MS) [62] liquid culture medium, added with 20 mM mannitol or 100 mM NaCl, and then planted for 7 days before collecting the samples. After a 21 d cultivation, rice seedlings were sampled for physiological, biochemical, and molecular assays. The expression levels of genes associated with phytic acid biosynthesis and stress response were measured.

### 4.8. Measurement of Inositol Triphosphate (IP_3_) and Phytic Acid (IP_6_) Content

Inositol triphosphate (IP_3_) was extracted by following the method of Campion et al. [63]. Approximately 2 g sample of fresh rice leaves was ground and blended in 3 mL 1 N HCl/methanol/chloroform (1.33/1/1). The homogenate was kept for 20 min at 4 °C, and then centrifuged at 3000× *g* for 20 min (4 °C). The organic phase (bottom) was discarded and the aqueous phase (top) was extracted again with a methanol/chloroform (*v*/*v*, 1/1). The top phase was dried in a rotary evaporator, and then suspended in 100 μL distilled water for determination of IP_3_ content using the commercial assay kit of enzyme-linked immunosorbent assay (ELISA) (Jiangsu Jingmei Biotechnology Co., Ltd., Yancheng, China) [64].

The accumulation of phytic acid (IP_6_) in rice leaves was extracted and measured by following the protocol as previously described [65]. About 0.3 g of rice leaves were ground and mixed in 10 mL of 0.2 M HCl. The homogenate was kept for 2 h at room temperature, and then centrifuged at 5000× *g* for 15 min. The supernatant was added with hydrated FeCl_3_ and then mixed with 6 mL of 5 M NaOH. After added with HNO_3_, the absorbance of the mixed solution was measured at 510 nm.

### 4.9. Hydrogen Peroxide (H_2_O_2_) Content Measurement

H_2_O_2_ accumulation was determined using the commercial assay kit for H_2_O_2_ content (Solarbio, Beijing, China) according to the method described by Zhang et al. [66] and Jiang et al. [67]. Approximately 10 mg of fresh rice leaves were homogenized and mixed with 500 μL acetone followed by centrifugation at 10,000× *g* for 8 min (4 °C). The supernatant was mixed with an equal volume of detection reagent of H_2_O_2_ by vortexing and incubated for 10 min at room temperature. The absorbance at 415 nm was determined, and the standard curve was used to assay the accumulation of H_2_O_2_.

### 4.10. Determination of Malondialdehyde (MDA)

The level of MDA was measured by following the method of Tang et al. [68]. About 100 mg rice leaves were homogenized and mixed with 10 mL trichloroacetic acid (*v*/*v*, 10%). The mixture was centrifuged for 15 min at 12,000× *g*. The reaction mix solution including 2 mL of thiobarbituric acid and 2 mL of extract was heated for 40 min at 95 °C, and then centrifuged once more for 15 min at 12,000× *g* after rapid cooling on ice. Finally, the absorbances were measured at 450, 532, and 600 nm.

### 4.11. Measurement of Free Proline (Pro)

The content of free proline was measured as previously reported [69]. About 1 g of rice tissue samples were ground with an aqueous solution of 3% (*v*/*v*) sulfosalicylic acid. After centrifugation at 37 °C at 12,000× *g* for 15 min, 2 mL of supernatant was added to the same volumes of glacial acetic acid and acidic ninhydrin. This mixture was heated at 95 °C for 40 min, cooled on ice, and then followed by the addition of 4 mL of toluene to extract the colored reaction product separated from the aqueous phase. The absorbance of the toluene phase at 520 nm was determined and the standard curve of proline was utilized to assay the accumulation of free proline.

### 4.12. Measurement of Anti-Oxidant Enzymes Activities

The activities of anti-oxidant enzymes, peroxidase (POD), catalase (CAT), and superoxide dismutase (SOD), were determined as reported [70,71,72,73] by using the commercial kits (Solarbio, Beijing, China). The anti-oxidant enzyme activities were standardized as follows: one unit of POD activity causes the absorbance to induce 0.01 at 470 nm per min; one unit of SOD activity causes the inhibition of photoreduction of 4-nitro blue tetrazolium chloride by 50%; one unit of CAT activity causes reduction of 1 nmol H_2_O_2_ per min.

### 4.13. Analysis of Gene Expression

The RNAprep Pure Plant Kit (Tiangen, Beijing, China) was used to extract the total RNA of fresh rice tissues and HiScript III 1st Strand cDNA Synthesis Kit (Vazyme Biotech Co., Ltd., Nanjing, China) was used to generate cDNA from total RNA by reverse transcription [74]. AceQ qPCR SYBR Green Master Mix (Vazyme, Nanjing, China) was used to perform quantitative real-time PCR (qRT-PCR) and primers used in this study for qRT-PCR are shown in Table S1. The levels of relative gene expression were performed by using the method of 2^−ΔΔCt^ [75] and the ACTIN gene (*Os03g0718100*) was utilized as the internal reference.

### 4.14. Statistical Analysis

Three replicates were executed for molecular, physiological, and biochemical assays, and six replicates were conducted for the measurements of dry biomass, plant height, and root length. The experimental data values are shown as the means standard error (SE) based on replications. The statistical analyses were completed by following the students’ *t*-test. The means were compared by analysis of variance (ANOVA), and the significant differences between means of groups were performed using Bonferroni post-tests.

## Data Availability

The data presented in this study are available in the article or supplementary material.

## Supplementary Materials

The following are available online at https://www.mdpi.com/2223-7747/10/1/23/s1, Figure S1: The predicted protein of mutant line is shown together with its WT one using the Clustal Omega Multiple Sequence Alignment (https://www.ebi.ac.uk/Tools/msa/clustalo/), Figure S2. Multiple sequence alignment of IPK1s, Figure S3. Relative expression level of phytic acid biosynthesis genes in Xidao #1 (WT) and its *OsIPK1* mutant line *osipk1_1*, Table S1. Primers used in this study.

## References

[1] Lott J.N.A., Ockenden I., Raboy V., Batten G.D. (2000). Phytic acid and phosphorus in crops seeds and fruits: A global estimate. Seed Sci. Res..

[2] Zhao H.J., Frank T., Tan Y.Y., Zhou C.G., Jabnoune M., Arpat A.B., Cui H.R., Huang J.Z., He Z.H., Poirier Y. (2016). Disruption of *OsSULTR3;3* reduces phytate and phosphorus concentrations and alters the metabolite profile in rice grains. New Phytol..

[3] Raboy V., Gerbasi P.F., Young K.A., Stoneberg S.D., Pickett S.G., Bauman A.T., Murthy P.P.N., Sheridan W.F., Ertlet D.S. (2000). Origin and seed phenotype of maize *low phytic acid 1-1* and *low phytic acid 2-1*. Plant Physiol..

[4] Kuwano M., Ohyama A., Tanaka Y., Mimura T., Takaiwa F., Yoshida K.T. (2006). Molecular breeding for transgenic rice with low phytic acid phenotype through manipulating myo inositol 3 phosphate synthase gene. Mol. Breed..

[5] Raboy V. (2009). Approaches and challenges to engineering seed phytate and total phosphorus. Plant Sci..

[6] Bohn L., Meyer A.S., Rasmussen S.K. (2008). Phytate: Impact on environment and human nutrition, a challenge for molecular breeding. J. Zhejiang Univ. Sci. B.

[7] Pilu R., Panzeri D., Gavazzi G., Rasmussen S.K., Consonni G., Nielsen E. (2003). Phenotypic, genetic and molecular characterization of a maize low phytic acid mutant (*lpa 241*). Theor. Appl. Genet..

[8] Guttieri M., Bowen D., Dorsch J.A., Raboy V., Souza E. (2014). Identification and characterization of a low phytic acid wheat. Crop Sci..

[9] Larson S.R., Rutger J.N., Young K.A., Raboy V. (2000). Isolation and genetic mapping of a non-lethal rice (*Oryza sativa* L.) *low phytic acid 1* mutation. Crop Sci..

[10] Liu Q.L., Xu X.H., Ren X.L., Fu H.W., Wu D.X., Shu Q.Y. (2007). Generation and characterization of low phytic acid germplasm in rice (*Oryza sativa* L.). Theor. Appl. Genet..

[11] Kim S.I., Andaya C.B., Newman J.W., Goyal S.S., Tai T.H. (2008). Isolation and characterization of a low phytic acid rice mutant reveals a mutation in the rice orthologue of maize MIK. Theor. Appl. Genet..

[12] Li W.X., Huang J.Z., Zhao H.J., Tan Y.Y., Cui H.R., Poirier Y., Shu Q.Y. (2014). Production of low phytic acid rice by hairpin RNA- and artificial microRNA-mediated silencing of *OsMIK* in seeds. Plant Cell Tiss. Org..

[13] Li W.X., Zhao H.J., Pang W.Q., Cui H.R., Poirier Y., Shu Q.Y. (2014). Seed-specific silencing of OsMRP5 reduces seed phytic acid and weight in rice. Transgenic Res..

[14] Suzuki M., Tanaka K., Kuwano M., Yoshida K.T. (2007). Expression pattern of inositol phosphate-related enzymes in rice (*Oryza sativa* L.): Implications for the phytic acid biosynthetic pathway. Gene.

[15] Stephens L.R., Irvine R.F. (1990). Stepwise phosphorylation of myo-inositol leading to myo-inositol hexakisphosphate in Dictyostelium. Nature.

[16] Brearley C.A., Hanke D.E. (1996). Metabolic evidence for the order of addition of individual phosphate este rs to the myo-inositol moiety of inositol hexakisphosphate in the duckweed *Spirodela polyrhiza* L.. Biochem. J..

[17] York J.D., Odom A.R., Murphy R., Ives E.B., Wente S.R. (1999). A phospholipase cdependent inositol polyphosphate kinase pathway required for efficient messenger RNA export. Science.

[18] Stevenson-Paulik J., Bastidas R.J., Chiou S.-T., Frye R.A., York J.D. (2005). Generation of phytate-free seeds in Arabidopsis through disruption of inositol polyphosphate kinases. Proc. Nat. Acad. Sci. USA.

[19] Ali N., Paul S., Gayen D., Sarkar S.N., Datta K., Datta S.K. (2013). Development of low phytate rice by RNAi mediated seed specific seed specific silencing of inositol 1,3,4,5,6-pentakisphosphate 2-kinase gene (*IPK1*). PLoS ONE.

[20] Yuan F.J., Zhao H.J., Ren X.L., Zhu S.L., Fu X.J., Shu Q.Y. (2007). Generation and characterization of two novel low phytate mutations in soybean (Glycine max L. Merr.). Theor. Appl. Genet..

[21] Yuan F.J., Zhu D.H., Tan Y.Y., Dong D.K., Fu X.J., Zhu S.L., Li B.Q., Shu Q.Y. (2012). Identification and characterization of the soybean IPK1 ortholog of a low phytic acid mutant reveals an exon-excluding splice-site mutation. Theor. Appl. Genet..

[22] Xiong L., Lee B., Ishitani M., Lee H., Zhang C., Zhu J.K. (2001). FIERY1 encoding an inositol polyphosphate 1-phosphatase is a negative regulator of abscisic acid and stress signaling in Arabidopsis. Genes Dev..

[23] DeWald D.B., Torabinejad J., Jones C.A., Shope J.C., Cangelosi A.R., Thompson J.E., Prestwich G.D., Hama H. (2001). Rapid accumulation of phosphatidylinositol 4, 5-bisphosphate and inositol 1, 4, 5-trisphosphate correlates with calcium mobilization in salt stressed Arabidopsis. Plant Physiol..

[24] Lu Y., Ye X., Guo R., Huang J., Wang W., Tang J., Tan L., Zhu J.K., Chu C., Qian Y. (2017). Genome-wide targeted mutagenesis in rice using the crispr/cas9 system. Mol. Plant.

[25] Jung C., Capistrano-Gossmann G., Braatz J., Sashidhar N., Melzer S. (2017). Recent developments in genome editing and applications in plant breeding. Plant Breed..

[26] Jiang M., Liu Y., Liu Y., Tan Y., Huang J., Shu Q. (2019). Mutation of inositol 1,3,4-trisphosphate 5/6-kinase6 impairs plant growth and phytic acid synthesis in rice. Plants-Basel.

[27] Stevenson J.M., Perera I.Y., Heilmann I.I., Persson S., Boss W.F. (2000). Inositol signaling and plant growth. Trends Plant Sci..

[28] Ziolkowski N., Grover A.K. (2010). Functional linkage as a direction for studies in oxidative stress: Alpha-adrenergic receptors. Can. J. Physiol. Pharmacol..

[29] Shi J., Wang H., Hazebroek J., Ertl D.S., Harp T. (2005). The maize *low-phytic acid 3* encodes a myo-inositol kinase that plays a role in phytic acid biosynthesis in developing seeds. Plant J..

[30] Khodakovskaya M., Sword C., Wu Q., Perera I.Y., Boss W.F., Brown C.S., Sederoff H.W. (2010). Increasing inositol (1, 4, 5)-trisphosphate metabolism affects drought tolerance, carbohydrate metabolism and phosphate-sensitive biomass increases in tomato. Plant Biotech. J..

[31] Yancey P.H., Clark M.E., Hand S.C., Bowlus R.D., Somero G.N. (1982). Living with water stress: Evolution of osmolyte systems. Science.

[32] Le Rudulier D., Strom A.R., Dandekar A.M., Smith L.T., Valentine R.C. (1984). Molecular biology of osmoregulation. Science.

[33] Hong Z., Lakkineni K., Zhang Z., Verma D.P. (2000). Removal of feedback inhibition of delta(1)-pyrroline-5-carboxylate synthetase results in increased proline accumulation and protection of plants from osmotic stress. Plant Physiol..

[34] Mittler R. (2002). Oxidative stress, antioxidants and stress tolerance. Trends Plant Sci..

[35] Apel K., Hirt H. (2004). Reactive oxygen species: Metabolism, oxidative stress, and signal transduction. Annu. Rev. Plant Biol..

[36] Graf E., Eaton J.W. (1990). Antioxidant functions of phytic acid. Free Radic. Biol. Med..

[37] Enrico D., Luciano G., Lucia C., Calogero P., Roberto P., Elena C., Erik N. (2009). Phytic acid prevents oxidative stress in seeds: Evidence from a maize (Zea mays L.) low phytic acid mutant. J. Exp. Bot..

[38] Du H., Liu L.H., You L., Yang M., He Y.B., Li X.H., Xiong L.Z. (2011). Characterization of an inositol 1,3,4-trisphosphate 5/6-kinase gene that is essential for drought and salt stress responses in rice. Plant Mol. Biol..

[39] Chittoor J.M., Leach J.E., White F.F. (1997). Differential induction of a peroxidase gene family during infection of rice by Xanthomonas oryzae pv. Oryzae. Mol. Plant-Microbe Interact..

[40] Hu H., You J., Fang Y., Zhu X., Qi Z., Xiong L. (2008). Characterization of transcription factor gene SNAC2 conferring cold and salt tolerance in rice. Plant Mol. Biol..

[41] Dubouzet J.G., Sakuma Y., Ito Y., Kasuga M., Dubouzet E.G., Miura S., Seki M., Shinozaki K., Yamaguchi-Shinozaki K. (2003). OsDREB genes in rice, Oryza sativa L. encode transcription activators that function in drought-, high-salt- and cold-responsive gene expression. Plant J..

[42] Hu H., Dai M., Yao J., Xiao B., Li X., Zhang Q., Xiong L. (2006). Overexpressing a NAM, ATAF, and CUC (NAC) transcription factor enhances drought resistance and salt tolerance in rice. Proc. Natl. Acad. Sci. USA.

[43] Xiang Y., Tang N., Du H., Ye H., Xiong L. (2008). Characterization of OsbZIP23 as a key player of the basic leucine zipper transcription factor family for conferring abscisic acid sensitivity and salinity and drought tolerance in rice. Plant Physiol..

[44] Hou X., Xie K., Yao J., Qi Z., Xiong L. (2009). A homolog of human skiinteracting protein in rice positively regulates cell viability and stress tolerance. Proc. Natl. Acad. Sci. USA.

[45] Wu G.T., Shu Q.Y., Xia Y.W. (1997). The biological effects of Pingyangmycin on rice. J. Zhejiang Agric. Univ..

[46] Shu Q.Y., Cui H.R., Ye G.Y., Wu D.X., Xia Y.W., Gao M.W., Altosaar I. (2002). Agronomic and morphological characterization of agrobacterium-transformed bt rice plants. Euphytica.

[47] Kaeppler S.M., Kaeppler H.F., Rhee Y. (2000). Epigenetic aspects of somaclonal variation in plants. Plant Mol. Biol..

[48] Lei Y., Lu L., Liu H.Y., Li S., Xing F., Chen L.L. (2014). CRISPR-P: A web tool for synthetic single-guide RNA design of CRISPR-system in plants. Mol. Plant.

[49] Lu H.P., Liu S.M., Xu S.L., Chen W.Y., Zhou X., Tan Y.Y., Huang J.Z., Shu Q.Y. (2017). CRISPR-S: An active interference element for a rapid and inexpensive selection of genome-edited, transgene-free rice plants. Plant Biotechnol. J..

[50] Xu R.F., Li H., Qin R.Y., Wang L., Li L., Wei P.C., Yang J.B. (2014). Gene targeting using the Agrobacterium tumefaciens-mediated CRISPR-Cas system in rice. Rice.

[51] Li W.X., Wu S.L., Liu Y.H., Jin G.L., Zhao H.J., Fan L.J., Shu Q.Y. (2016). Genome-wide profiling of genetic variation in Agrobacterium-transformed rice plants. J. Zhejiang Univ. Sci. B.

[52] Zhang H.L., Huang J.Z., Chen X.Y., Tan Y.Y., Shu Q.Y. (2014). Competitive amplification of differentially melting amplicons facilitates efficient genotyping of photoperiod-and temperature-sensitive genic male sterility in rice. Mol. Breed..

[53] Li W.L., Xu B.B., Song Q.J., Liu X.M., Xu J.M., Brookes P.C. (2014). The identification of ‘hotspots’ of heavy metal pollution in soil-rice systems at a regional scale in eastern China. Sci. Total Environ..

[54] Li S., Liu S.M., Liu Y.H., Lu H.P., Tan Y.Y., Huang J.Z., Wei P.C., Shu Q.Y. (2018). HRM-facilitated rapid identification and genotyping of mutations induced by CRISPR/Cas9 mutagenesis in rice. Crop Breed. Appl. Biotechnol..

[55] Liu W.Z., Xie X.R., Ma X.L., Li J., Chen J.H., Liu Y.G. (2015). DSDecode: Aweb-based tool for decoding of sequencing chromatograms for genotyping of targeted mutations. Mol. Plant.

[56] Chen P.S., Toribara T.Y., Warner H. (1956). Micro determination of phosphorous. Anal. Chem..

[57] Wilcox J.R., Premachandra G.S., Young K.A., Raboy V. (2000). Isolation of high seed inorganic P, low-phytate soybean mutants. Crop Sci..

[58] Raboy V., Young K.A., Dorsch J.A., Cook A. (2001). Genetics and breeding of seed phosphorus and phytic acid. J. Plant Physiol..

[59] McKie V.A., McCleary B.V.A. (2016). Novel and rapid colorimetric method for measuring total phosphorus and phytic acid in foods and animal feeds. J. AOAC Int..

[60] Hu L.F., McBride M.B., Cheng H., Wu J.J., Shi J.C., Xu J.M., Wu L.S. (2011). Root-induced changes to cadmium speciation in the rhizosphere of two rice (*Oryza sativa* L.) genotypes. Environ. Res..

[61] Meng J., Zhong L.B., Wang L., Liu X.M., Tang C.X., Chen H.L., Xu J.M. (2018). Contrasting effects of alkaline amendments on the bioavailability and uptake of Cd in rice plants in a Cd-contaminated acid paddy soil. Environ. Sci. Pollut. R..

[62] Murashige T., Skoog F. (1962). A revised medium for rapid growth and bio assays with tobacco tissue cultures. Physiol. Plantarum.

[63] Campion B., Sparvoli F., Doria E., Tagliabue G., Galasso I., Fileppi M., Bollini R., Nielsen E. (2009). Isolation and characterization of an *lpa* (*low phytic acid*) mutant in common bean (*Phaseolus vulgaris* L.). Theor. Appl. Genet..

[64] Jiang M., Liu Y., Li R., Zheng Y., Fu H., Tan Y., Moller I.M., Fan L., Shu Q., Huang J. (2019). A suppressor mutation partially reverts the xantha trait via lowered methylation in the promoter of genomes uncoupled 4 in rice. Front. Plant Sci..

[65] Miller G.A., Youngs V.L., Oplinger E.S. (1980). Environmental and cultivar effects on oat phytic acid concentration. Cereal Chem..

[66] Zhang H., Liu Y., Wen F., Yao D., Wang L., Guo J., Nan L., Zhang A., Tana M., Jiang M. (2014). A novel rice C_2_H_2_-type zinc finger protein, ZFP36, is a key player involved in abscisic acid-induced antioxidant defence and oxidative stress tolerance in rice. J. Exp. Bot..

[67] Jiang M., Jiang J., Li S., Li M., Tan Y.Y., Song S.Y., Shu Q.Y., Huang J.Z. (2020). Glutamate alleviates cadmium toxicity in rice via suppressing cadmium uptake and translocation. J. Hazard. Mater..

[68] Tang L., Cai H., Ji W., Luo X., Wang Z., Wu J., Wang X., Cui L., Wang Y., Zhu Y. (2013). Overexpression of GsZFP1 enhances salt and drought tolerance in transgenic alfalfa (*Medicago sativa* L.). Plant Physiol. Bioch..

[69] He J., Duan Y., Hua D., Fan G., Wang L., Liu Y., Chen Z., Han L., Qu L.J., Gong Z. (2012). DEXH Box RNA Helicase-mediated mitochondrial reactive oxygen species production in Arabidopsis mediates crosstalk between abscisic acid and auxin signaling. Plant Cell.

[70] Aebi H. (1984). Catalase in vitro. Method Enzymol..

[71] Zhang W., Zhang F., Raziuddin R., Gong H., Yang Z., Lu L., Ye Q., Zhou W. (2008). Effects of 5-aminolevulinic acid on oilseed rape seedling growth under herbicide toxicity stress. J. Plant Growth Regul..

[72] Hossain M.A., Hasanuzzaman M., Fujita M. (2010). Up-regulation of antioxidant and glyoxalase systems by exogenous glycinebetaine and proline in mung bean confer tolerance to cadmium stress. Physiol. Mol. Biol. Plants.

[73] Jiang M., Wang J.X., Rui M.M., Yang L.J., Shen J., Chu H.W., Song S.Y., Chen Y. (2020). OsFTIP7 determines metallic oxide nanoparticles response and tolerance by regulating auxin biosynthesis in rice. J. Hazard. Mater..

[74] Jiang M., Wu X.J., Song Y., Shen H.Z., Cui H.R. (2020). Effects of OsMSH6 mutations on microsatellite stability and homeologous recombination in rice. Front. Plant Sci..

[75] Livak K.J., Schmittgen T.D. (2001). Analysis of relative gene expression data using real-time quantitative PCR and the 2(-delta delta c (t)) method. Methods.

