# Supplementary material for: An Inositol 1,3,4,5,6-Pentakisphosphate 2-Kinase 1 Mutant with a 33-nt Deletion Showed Enhanced Tolerance to Salt and Drought Stress in Rice
Source: Plants (Basel). 2020 Dec 24;10(1):23. doi: 10.3390/plants10010023 (PMC7824669; doi:10.3390/plants10010023)
Supplement: Supplementary file 1 [file plants-10-00023-s001.pdf]

```
WT:        MEVVLHEGDAKDWVYKGEGAANLILSYTGSSPSMLGKVLRVKKILKDKGQPAPNCIVFSS   60
Osipk1_1:  MEVVLHEGDAKDWVYKGEGAANLILSYTGSSPSMLGKVLRVKKILKDKGQPAPNCIVFSS   60
Osipk1_2:  MEVVLHEGDAKDWVYKGEGAANLILSYTGSSPSMLGKVLRVKKILKDKGQPAPNCIVFSS   60
Osipk1_3:  MEVVLHEGDAKDWVYKGEGAANLILSYTGSSPSMLGKVLRVKKILKDKGQPAPNCIVFSS   60
           ************************************************************

WT:        HEEHLWGKIPGLLESVKNDCLPQAYATIVMSQHLGANHVDGGVRVRVSKNFFELAGKNVL   120
Osipk1_1:  HEEH-----------VKNDCLPQAYATIVMSQHLGANHVDGGVRVRVSKNFFELAGKNVL   109
Osipk1_2:  HEEHLWGKIPGCWNLLKMIACHKPMLQLL-------------------------------   89
Osipk1_3:  HEEHLWGKIPGVGIC---------------------------------------------   75
           ****

WT:        DNRPAWRVNASAIDAGADSALLISDHTLFSGNPRGSSCIAVEIKAKCGFLPSSEYISKEN   180
Osipk1_1:  DNRPAWRVNASAIDAGADSALLISDHTLFSGNPRGSSCIAVEIKAKCGFLPSSEYISKEN   169
Osipk1_2:  ------------------------------------------------------------   89
Osipk1_3:  ------------------------------------------------------------   75

WT:        SIKKQVTRYKMHQHLKFHLGEISKTSEYDPLDLFSGSKERIHMAIKSFFSTPQNNFRIFV   240
Osipk1_1:  SIKKQVTRYKMHQHLKFHLGEISKTSEYDPLDLFSGSKERIHMAIKSFFSTPQNNFRIFV   229
Osipk1_2:  ------------------------------------------------------------   89
Osipk1_3:  ------------------------------------------------------------   75

WT:        DGSLVFGGMGGGADSVHPNETEKCLEDLSKVTGLQLSDFIELLSEAIFKSGVLGKLLATQ   300
Osipk1_1:  DGSLVFGGMGGGADSVHPNETEKCLEDLSKVTGLQLSDFIELLSEAIFKSGVLGKLLATQ   289
Osipk1_2:  ------------------------------------------------------------   89
Osipk1_3:  ------------------------------------------------------------   75

WT:        KLDDHDIEGAIHLYYNIISQPCLVCKSITDTELLRKYSTLHSLPLDKSEKIVRDFLISAT   360
Osipk1_1:  KLDDHDIEGAIHLYYNIISQPCLVCKSITDTELLRKYSTLHSLPLDKSEKIVRDFLISAT   349
Osipk1_2:  ------------------------------------------------------------   89
Osipk1_3:  ------------------------------------------------------------   75

WT:        AKDCSLMISFRPRQSGTTDSEYDSVFLDSVNQSYDYKAYFIDLDVKPLDKMVHYFKLDQK   420
Osipk1_1:  AKDCSLMISFRPRQSGTTDSEYDSVFLDSVNQSYDYKAYFIDLDVKPLDKMVHYFKLDQK   409
Osipk1_2:  ------------------------------------------------------------   89
Osipk1_3:  ------------------------------------------------------------   75

WT:        IVNFYTRNGEVGGDPRDPPKGCGPR   445
Osipk1_1:  IVNFYTRNGEVGGDPRDPPKGCGPR   434
Osipk1_2:  -------------------------   89
Osipk1_3:  -------------------------   75
```

**Figure S1.** The predicted protein of mutant line is shown together with its WT one using the Clustal Omega Multiple Sequence Alignment (https://www.ebi.ac.uk/Tools/msa/clustalo/).

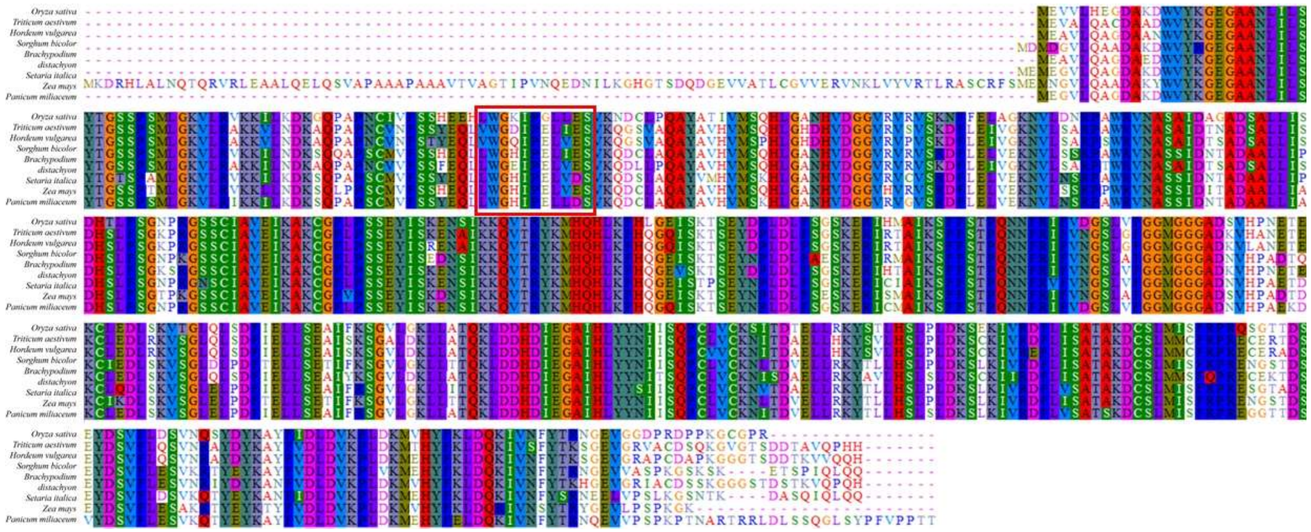

**Figure S2.** Multiple sequence alignment of IPK1s. All the datas are from RAP-DB (http://rapdb.dna.affrc.go.jp/) and Gramene (http://www.gramene.org/). Analysis of IPK1 sequences from nine organisms using BioEdit software. Identical amino acid residues are boxed in same color. Short lines indicate gaps introduced during alignment. The mutant site of OsIPK1 is marked by a red box.

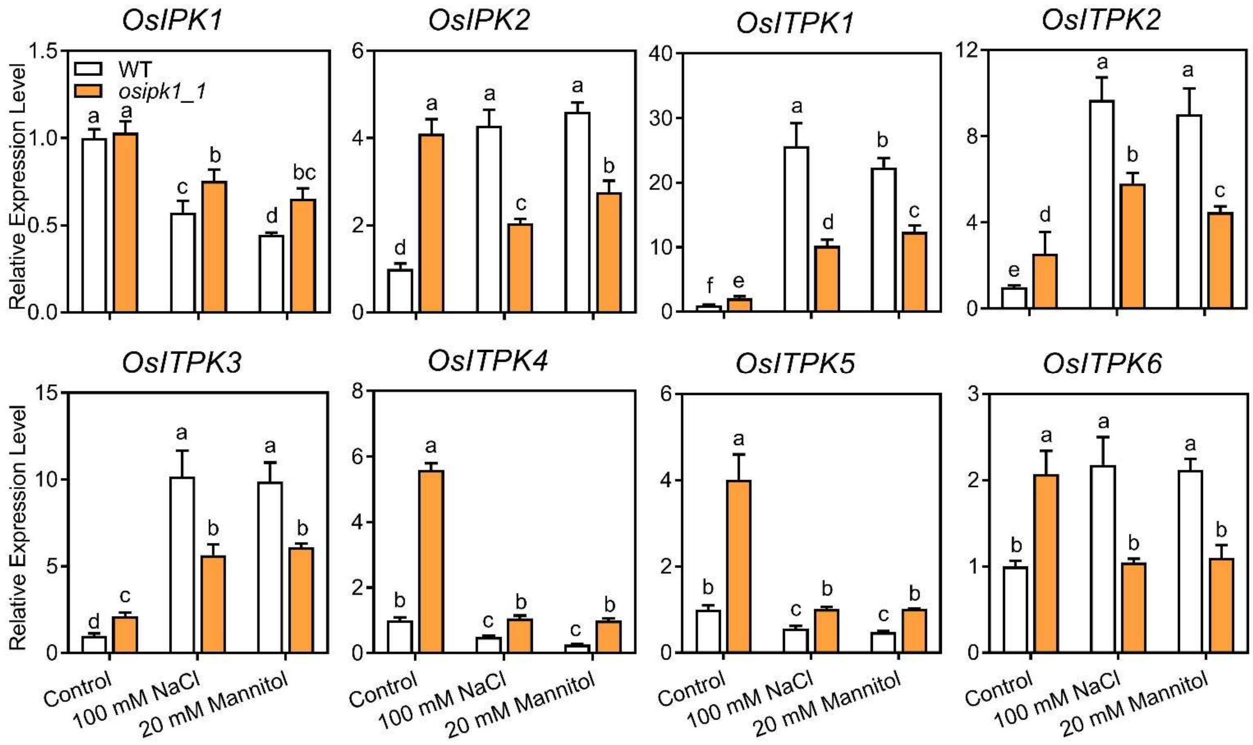


**Figure S3.** Relative expression level of phytic acid biosynthesis genes in Xidao #1 (WT) and its *OsIPK1* mutant line *osipk1_1*. All analyses were performed with three replicates. Error bars represent standard error. The different letters show the significant difference at a probability of $p < 0.05$.

**Table 1.** Primers used in this study.

| Primer Name | Primer Sequence (5'→3') |
|---|---|
| C1-F | GGCAGGCAAGATCCCAGGATTGT |
| C1-R | AAACACAATCCTGGGATCTTGCC |
| HygR-F | AGAAGAAGATGTTGGCGACCT |
| HygR-R | GTCCTGCGGGTAAATAGCT |
| P1-F | GGCAAGGTTCTGCGAGTC |
| P1-R | CCGCCATCAACATGATTGG |
| qRT-OsIPK1F | TGTGTTTCTACAAGTGTGCATG |
| qRT-OsIPK1R | CAGATCACAGATTCACAGCAAG |
| qRT-OsIPK2-F | TTTGAAAGAGGAAACTTCACCG |
| qRT-OsIPK2-R | GAATCCCATGGTTTTAAAGGGG |
| qRT-OsITPK1F | CCAGGACGATATTAAGAACA |
| qRT-OsITPK1R | GGAACTCAGTGAACGATT |
| qRT-OsITPK2-F | GGCGAGGAAGAAGGGAATTAAT |
| qRT-OsITPK2-R | AAATGGGCCTTGTTCTGCAA |
| qRT-OsITPK3-F | AGTTATCTCTTGCTTATGA |
| qRT-OsITPK3-R | CCAACAATGTAGACCTTA |
| qRT-OsITPK4-F | CCGACCCAGCGTTTGAAC |
| qRT-OsITPK4-R | TTGCTTGGCTTCATTGATTATTGTT |
| qRT-OsITPK5-F | AATCCTTTCTCCTAACTCCTTTC |
| qRT-OsITPK5-R | TCTCTTGGCTGCTCTAACTA |
| qRT-OsITPK6-R | GCGACAACATTCATACTAAT |
| qRT-OsITPK6-R | CTAAGTGACAAGCAACCT |
| qRT-OsPOX8.1-F | CTGCTCCAAAGTGAACTAATAATTAAGTAAAG |
| qRT-OsPOX8.1-R | CCCAGCCTTATTCCCAAATTT |
| qRT-OsPOX22.3-F | CAGCTGCTCCAAGGTGAACTC |
| qRT-OsPOX22.3-R | AGATTTGCTTCCAGCAACGAA |
| qRT-OsP5CS-F | GCTGACATGGATATGGCAAAAC |
| qRT-OsP5CS-R | GTAAGGTCTCCATTGCATTGCA |
| qRT-OsRab16D-F | CGGGTAAACAATAAAGTCGTGATG |
| qRT-OsRab16D-R | GCGCACTTACATACAGTGCTACGT |
| qRT-OsGDSL-F | TCATCGTACAGCAGTTCAT |
| qRT-OsGDSL-R | TTGTTGGTCGAGCTTGAG |
| qRT-OsbZIP23-F | GGAGCAGCAAAAGAATGAGG |
| qRT-OsbZIP23-R | GGTCTTCAGCTTCACCATCC |
| qRT-OsSNAC1-F | CATGGTCCCGTTCTGAGGTG |
| qRT-OsSNAC1-R | CACACGTTGCAGCATCGATC |
| qRT-DREB1A-F | AGGACTTCTTCCGGCGCCGCCT |
| qRT-DREB1A-R | TCGTCGTCGTCGGTGCGTGGGGT |
| qRT-OsActin-F | TGGCATCTCTCAGCACATTCC |
| qRT-OsActin-R | TGCACAATGGATGGGTCAGA |
